# Modeling blood diseases with human induced pluripotent stem cells

**DOI:** 10.1242/dmm.039321

**Published:** 2019-06-04

**Authors:** Maria Georgomanoli, Eirini P. Papapetrou

**Affiliations:** Department of Oncological Sciences, Icahn School of Medicine at Mount Sinai, New York, NY 10029, USA; Tisch Cancer Institute, Icahn School of Medicine at Mount Sinai, New York, NY 10029, USA; Black Family Stem Cell Institute, Icahn School of Medicine at Mount Sinai, New York, NY 10029, USA; Department of Medicine, Icahn School of Medicine at Mount Sinai, New York, NY 10029, USA

**Keywords:** iPSC generation, Hematopoietic differentiation, Genetic blood disorders, Monogenic disorders, Myeloid malignancies

## Abstract

Induced pluripotent stem cells (iPSCs) are derived from somatic cells through a reprogramming process, which converts them to a pluripotent state, akin to that of embryonic stem cells. Over the past decade, iPSC models have found increasing applications in the study of human diseases, with blood disorders featuring prominently. Here, we discuss methodological aspects pertaining to iPSC generation, hematopoietic differentiation and gene editing, and provide an overview of uses of iPSCs in modeling the cell and gene therapy of inherited genetic blood disorders, as well as their more recent use as models of myeloid malignancies. We also discuss the strengths and limitations of iPSCs compared to model organisms and other cellular systems commonly used in hematology research.

## Abstract (precis)

**Summary:** This Review discusses the applications of human iPSCs as preclinical models of cell and gene therapy for inherited monogenic blood disorders, as well as their more recent use as models of myeloid malignancies.

## Introduction

The study of blood diseases has historically been at the forefront of biomedical research, mainly because of the accessibility of hematopoietic tissue from the peripheral blood (PB) or the bone marrow (BM) of patients and healthy individuals. Hematology research has been mainly fueled by two material sources, individually imperfect and complementary to each other: primary human cells and model systems. More prominent among the latter are mouse models and immortalized cell lines established from human leukemias (Barretina et al., 2012; Gould et al., 2015). Other model organisms frequently used in hematology research include the zebrafish (Avagyan and Zon, 2016) and large animal models, such as the dog and non-human primates (Larochelle and Dunbar, 2013; Trobridge and Kiem, 2010).

Since the emergence of induced pluripotent stem cell (iPSC) technology, a little over a decade ago, iPSC models have held the potential to bridge these two worlds – primary patient cells and conventional disease models – by affording direct relevance to human disease, being patient-derived, while enabling precise, controlled and scalable experiments that were hitherto only possible using traditional model systems. Here, we review the use of iPSC models in the study of blood diseases, mainly encompassing two areas: the modeling of gene and cell therapy of inherited monogenic disorders, and the modeling of myeloid malignancies.

## Practical considerations of iPSC disease modeling

### Derivation of human iPSCs

iPSCs are cells derived from somatic cells through a process of reprogramming, which converts them to a pluripotent stem cell (PSC) state, resembling that of embryonic stem cells (ESCs), the prototypical PSCs (Box 1, Overview of methods for derivation of human iPSCs). The process of reprogramming to pluripotency silences the somatic cell gene expression program and activates pluripotency regulatory networks that sustain a self-renewing state (Jaenisch and Young, 2008). Thus, iPSCs can be grown as cell lines indefinitely, while maintaining pluripotency, i.e. the ability to differentiate into all the cell types of the human body. The generation of iPSCs was pioneered by Shinya Yamanaka in 2006, first in murine and subsequently in human cells (Takahashi and Yamanaka, 2006; Takahashi et al., 2007; Yu et al., 2007). The generation of iPSCs is nowadays a fairly mainstream procedure practiced by investigators worldwide. Numerous disease models have been developed with iPSC technology and genetic blood disorders were among the first to be modeled with patient-derived iPSCs. Box 1 summarizes the methodological aspects of iPSC derivation and the quality control of established iPSC lines.

**Box 1. Overview of methods for derivation of human iPSCs**

**Reprogramming factors (RFs)**

Reprogramming of somatic cells to pluripotency requires the transient forced expression of a combination of typically four or more ‘reprogramming factors’. The combination most widely used is the ‘Yamanaka factors’ OCT4, SOX2, KLF4 and MYC (OSKM), originally identified through a screen of 24 factors expressed in pluripotent cells (Takahashi et al., 2007; Takahashi and Yamanaka, 2006). Although omitting MYC or one or two more factors (with the exception of OCT4) is still sufficient for reprogramming in some cases, minimal factor cocktails generally work only with specific starting cell types and with low reprogramming efficiencies (Nakagawa et al., 2008; Huangfu et al., 2008b). An alternative combination is the ‘Thomson factors’ OCT4, SOX2, NANOG and LIN28, derived through an independent screen of candidate genes (Yu et al., 2007). The union of both cocktails, yielding six RFs (OCT4, KLF4, SOX2, MYC, LIN28 and NANOG), is sometimes used to enhance reprogramming efficiency (Yu et al., 2009; Tanabe et al., 2013).

A variety of additional genes and chemicals have been shown to boost reprogramming efficiency and are often referred to as reprogramming ‘enhancers’ (Takahashi and Yamanaka, 2016). Examples include genes with known roles in pluripotency, such as undifferentiated embryonic cell transcription factor 1 (*UTF1*), sal-like protein 4 (*SALL4*) and *Tbx3* (Zhao et al., 2008; Takahashi et al., 2007; Han et al., 2010), chromatin modifiers such as histone demethylases (Wang et al., 2011a), viral oncoproteins such as SV40T and the catalytic subunit of the human telomerase (hTERT) (Park et al., 2008b; Mali et al., 2008), and microRNAs (Judson et al., 2009). Inhibition of p53 enhances reprogramming efficiency, and an shRNA against p53 is a common addition to the reprogramming cocktail (Utikal et al., 2009; Marión et al., 2009; Li et al., 2009b; Kawamura et al., 2009; Hong et al., 2009; Banito et al., 2009). Small molecules and chemicals that can boost reprogramming include the histone deacetylase inhibitor valproic acid (VPA), the DNA methyltransferase inhibitors 5-azacytidine and trichostatin A (Huangfu et al., 2008b, 2008a), MEK and GSK pathway inhibitors (Li et al., 2009c, 2011b; Shi et al., 2008; Silva et al., 2008), butyrate (Liang et al., 2010; Mali et al., 2010) and vitamin C (Chen et al., 2013; Esteban et al., 2010; Wang et al., 2011a). Furthermore, fusing the VP16 transactivation domain to the classic RFs to increase their transcriptional activation potency (Wang et al., 2011b; Hammachi et al., 2012) or culture in hypoxic conditions (Yoshida et al., 2009) are additional strategies that have been employed towards enhancing the efficiency of reprogramming.

**Starting cell type**

Theoretically, any somatic cell type can be reprogrammed to pluripotency, provided that it can divide in culture, as cell division is necessary for resetting the epigenome to silence somatic gene expression and activate the pluripotency program (Guo et al., 2014; Hanna et al., 2009; Ruiz et al., 2011). In the modeling of inherited genetic diseases, any cell type that can be obtained from patients can be used for iPSC derivation, as they all contain the disease-causing mutations. In these cases, the choice of cell type is mainly directed by availability, accessibility of tissue and ease of tissue processing and culture. Thus, the two most common cell sources are skin fibroblasts and peripheral blood (PB) cells, with others less commonly used including bone marrow (BM) stromal cells (Papapetrou et al., 2011), keratinocytes (Aasen et al., 2008), adipocytes (Aoki et al., 2010; Sugii et al., 2010), urinary epithelial cells obtained from urine specimens (Park et al., 2015), amniotic fluid cells (Zhao et al., 2010; Li et al., 2009a) and fibroblasts from sources other than the dermis. In contrast, in the modeling of diseases caused by mutations in somatic cells and not in the germline – like cancer – the cell type for reprogramming is restricted to the cell-of-origin of the disease and its descendants. In the case of myeloid malignancies that we discuss in the main article, the cells that bear the cancer-associated mutations are found in hematopoietic tissues of patients, namely the BM and PB. The BM and PB contain a variety of hematopoietic cell types and reprogramming can be initiated with either total unfractionated mononuclear cells or specific cell types, most commonly hematopoietic stem/progenitor cells (HSPCs), T lymphocytes or erythroblasts. These can be either prospectively isolated or – more commonly – preferentially expanded from the bulk cell population by means of stimulation with appropriate growth factors, cytokines or stimulatory signals. For example, T cells can be stimulated to proliferate with lipopolysaccharide (LPS) or CD3/CD28 ligands (Themeli et al., 2013), and HSPCs and erythroblasts can be outgrown from either purified CD34^+^ HSPCs or total mononuclear cells with early-acting cytokines (FL, SCF, IL-3, TPO and others) or erythroblast-stimulating cytokines (SCF, EPO and others), respectively (Kotini et al., 2017).

**Delivery methods**

The first generation of delivery methods to introduce the RFs into cells were γ-retroviral and lentiviral vectors. These vectors randomly integrate the transgenes into the host genome and have the advantage of efficiently transducing a variety of somatic cell types. At the same time, they confer the risk of insertional mutagenesis if the viral promoters/enhancers activate oncogenes in the vicinity of the integration site (Mitchell et al., 2004; Schröder et al., 2002) and the risk of incomplete silencing or reactivation after initial silencing of the RF transgenes (Okita et al., 2007). In an effort to minimize the number of viral integrations and temporally contain RF expression, researchers developed polycistronic lentiviral vectors encoding all four factors in a single vector (linked by 2A peptides and IRES signals), doxycycline-inducible vectors and excisable vectors (Hockemeyer et al., 2008; Papapetrou et al., 2011; Sommer et al., 2009). The latter consisted of either Cre/loxP systems or transposon/transposase systems, such as piggyBac (Sommer et al., 2009; Woltjen et al., 2009; Xie et al., 2014; Yusa et al., 2009; Papapetrou et al., 2011). Subsequently, integration-free systems were developed, consisting mainly of non-integrating DNA vectors, such as adenoviral vectors (Stadtfeld et al., 2008), conventional plasmids (Okita et al., 2010), oriP/EBNA1 episomes (Yu et al., 2009) and minicircles (Narsinh et al., 2011). Finally, DNA-free methods based on RNA-mediated delivery by Sendai virus-based vectors (Fusaki et al., 2009; Nishimura et al., 2011) and repeated transfection of modified mRNAs (Warren et al., 2010), or based on protein delivery using recombinant proteins or cell extracts (Cho et al., 2010; Kim et al., 2009; Zhou et al., 2009) were also developed. Of all these, three methods confer sufficient ease and efficiency and remain in wide use today: episomes, mRNA transfection and Sendai viruses. It should be noted that mRNA transfection is only appropriate for cells grown in adherent conditions, which excludes hematopoietic lineages.

**Reprogramming culture**

As iPSCs are, in most practical aspects, identical to hESCs, researchers readily adapted the culture conditions originally developed for hESCs to the culture of iPSCs. Once iPSC lines are established, they are commonly grown as colonies on layers of mitotically inactivated mouse embryonic fibroblasts (MEFs) and passaged manually or enzymatically as aggregates (Thomson et al., 1998). The addition of Rho kinase (Rock) inhibitor in the culture media enabled the culture of hPSCs as single cells (Watanabe et al., 2007), and the development of extracellular matrices (such as Matrigel) enabled their growth in feeder-free conditions. Various investigators implemented several modifications to hPSC culture (Chen et al., 2014). Variations in culture conditions during induction of reprogramming are also practiced, but all generally involve a gradual transition from media and culture conditions appropriate for the starting cell type to those of hPSCs.

Thus, induction of reprogramming can be performed in normoxic or hypoxic conditions, in the absence or presence of chemicals, such as VPA, vitamin C and others discussed earlier, and on feeder-free conditions or feeders, which can be MEFs or human dermal fibroblasts. Clonal iPSC lines are most often established by manually picking colonies that form over time, which can be identified by their characteristic morphology and colony shape upon visual inspection under a light microscope. These colonies are candidate iPSCs pending further characterization and can, thereafter, be passaged, expanded and cryopreserved using standard hPSC culture procedures.

**Reprogramming efficiency**

Reprogramming is a stochastic and inefficient process. Only a small minority of cells that receive and express the RFs accomplish full reprogramming to iPSCs, typically over a 2 to 3-week-long induction culture. The reprogramming efficiency is generally lower than 0.1%. The barriers to reprogramming are imperfectly understood, but likely involve epigenetic barriers, senescence, DNA damage and potentially many additional factors. The determinants of reprogramming efficiency are also incompletely understood. Some known critical factors include the proliferation rate of the cells in culture, the age of the individual they were derived from and abnormal genetic and epigenetic states. As the most striking example of the latter, malignant cells generally reprogram with low efficiencies, as is further discussed in the main text.

**Ascertaining the PSC state**

Once new iPSC lines are established, some quality control steps are generally practiced, first to document stemness and pluripotency. Although hPSCs have characteristic morphology that is a strong predictor of true iPSCs, the battery of assays commonly used and recommended to document an hPSC state includes pluripotent marker immunostains (alkaline phosphatase, NANOG or cell-surface markers such as TRA-1-81, TRA-1-60 and SSEA3), detection of gene expression and DNA methylation states characteristic of hPSCs and, critically, the ascertainment of the capacity to generate cells of all three embryonic germ layers (ectoderm, endoderm and mesoderm) *in vitro* in differentiation cultures and *in vivo* in teratomas grown in immunodeficient mice (De Los Angeles et al., 2015). Teratoma formation has served as the gold-standard assay of human pluripotency, but has many drawbacks as it is time-consuming, not quantitative and uses animals. Thus, efforts towards its substitution with bioinformatic algorithms based on gene expression or gene regulatory networks – such as Pluritest (Muller et al., 2011), CellNet (Cahan et al., 2014), scorecard (Bock et al., 2011) and Teratoscore (Avior et al., 2015) – as well as with more standardized commercially available assays of *in vitro* differentiation into all germ layers, are underway.

**Additional quality control**

The characterization of newly established iPSC lines invariably also includes tests of genome integrity. Even though early reports suggested that reprogramming itself may introduce genomic instability, it was later shown that effectively all genetic lesions identified in iPSC lines are pre-existing in the somatic cells from which they are derived (Abyzov et al., 2012; Cheng et al., 2012; Young et al., 2012). For most research applications, exclusion of gross abnormalities by G-banding and/or comparative genomic hybridization array is generally sufficient. iPSC generation for therapeutic applications obviously requires higher resolution testing of genome integrity. These standards are under discussion by the scientific community and will likely include tests of the highest stringency, such as whole genome sequencing. Additional tests can be performed to ascertain the provenance of iPSC lines from specific individuals using DNA fingerprinting approaches and/or detection of specific disease-related mutations.

**Appropriate controls**

iPSC disease modeling is unique among human patient-based models in that it affords unlimited opportunities for well-controlled experiments through the derivation of appropriate control iPSC lines. The exemplary controls are isogenic lines that are generated by gene editing and are discussed in detail in the main text. Alternative controls, albeit less stringent, include: (1) Genetically matched normal iPSC lines that can be derived from healthy cells of the same patient in cases of somatic, but not inherited, diseases, such as cancer. These are not isogenic, as they typically differ in more than one driver and/or passenger mutations. (2) Normal iPSCs derived from related or unrelated healthy donors. It should be noted that non-isogenic controls require larger numbers of cell lines for reliable experiments to capture any donor-related genetic sources of phenotypic variation.

### Genetic engineering of human iPSCs to develop isogenic models

The possibility to generate isogenic pairs is a major asset of iPSC disease modeling and enormously important for robust and reproducible experiments. Isogenic lines can be generated either by introducing specific disease-causing or disease-associated mutations in normal iPSCs or by correcting them in patient-derived iPSCs or, ideally, through both approaches. Because of their clonal growth and unlimited expansion, iPSCs are highly amenable to precise genetic engineering (Hockemeyer and Jaenisch, 2016). Human PSCs (hPSCs) were believed to be refractory to genetic modification, particularly to homologous recombination (HR); however, with improved single-cell culture conditions and more efficient genetic engineering methods, their genetic modification is now a routine practice (Watanabe et al., 2007).

Early genetic engineering approaches used HR, mediated through donor templates containing very long homology arms with very low efficiencies but, in more recent years, the advent of site-specific nucleases, and the CRISPR-Cas9 system in particular, has hugely facilitated and streamlined gene editing. Site-specific nucleases can be directed to introduce double-strand breaks (DSBs) at specific DNA sequences. Their specificity is determined through DNA–protein interactions in the case of homing endonucleases or meganucleases, zinc-finger nucleases (ZFNs) and transcription activator-like effector nucleases (TALENs), or through DNA–RNA base pairing in the case of CRISPR-Cas9 (Kim and Kim, 2014). Because targeting of the CRISPR system to a specific sequence is mediated through a guide RNA (gRNA), this system can be easily engineered to recognize specific sites and has thus found widespread use. DSBs can be repaired by the host cell through two distinct mechanisms that can be leveraged for genetic engineering, nonhomologous end-joining (NHEJ) or homology-directed repair (HDR). NHEJ is imprecise and typically introduces small insertions and deletions (indels), which often cause frameshifts to the coding sequence (at a theoretical two in three chance), resulting in functional knockout of a protein-coding gene. Conversely, HDR can be used to introduce a precise genetic modification, such as a point mutation or insertion, through the co-delivery of a donor DNA template (Cox et al., 2015; Sterneckert et al., 2014). The CRISPR-Cas9 system can also facilitate the engineering of large-scale genetic lesions often found in cancers, such as chromosomal deletions, inversions and translocations (Kotini et al., 2015; Maddalo et al., 2014; Schneidawind et al., 2018).

CRISPR-mediated gene editing requires the delivery of three components, the Cas9 nuclease, the gRNA and a donor template, in the case of HDR-mediated editing. This can be done through transfection of plasmid DNA or *in vitro* transcribed mRNAs, viral vectors or ribonucleoproteins (Kim et al., 2014). Importantly, because the goal is to derive edited clonal lines and iPSCs can be readily single-cell cloned and expanded, modest gene editing efficiencies are sufficient for most applications. The versatility and ease of the CRISPR-Cas9 system is somewhat constrained by the requirement for a specific proto-spacer adjacent motif (PAM) next to the target site (Sternberg et al., 2014). The most widely used CRISPR-Cas9 system is derived from *Streptococcus* *pyogenes* and requires a 5′-NGG PAM sequence, but the discovery of several Cas9 orthologs with different PAM requirements extends the repertoire of target sites (Cong et al., 2013; Jinek et al., 2012; Mali et al., 2013).

When using nuclease-mediated gene editing, researchers must consider the possibility for off-target effects and induction of translocations. Several techniques for the prediction and detection of off-target effects and strategies to develop nucleases with reduced off-targeting have been reported (Tsai and Joung, 2016; Tycko et al., 2016), including modifications of the gRNA (Cho et al., 2014; Fu et al., 2014; Kim et al., 2016) or engineering of the Cas9 (Guilinger et al., 2014; Ran et al., 2013a,b; Shen et al., 2014; Slaymaker et al., 2016; Tsai et al., 2014). Although therapeutic applications will require high standards of detection and exclusion of cells with off-target effects, one practical way to offset the potential phenotypic effects due to off-targeting is to derive isogenic lines using more than one gRNA, as different gRNAs will have different on- and off-target sites. A recent alternative to gene editing through DSB-facilitated HDR is the use of base editors that introduce site-specific modification of DNA bases (Rees and Liu, 2018). These are engineered chimeric proteins composed of a DNA recognition module (commonly catalytically inactive Cas9) and a catalytic domain capable of deaminating a cytidine or adenine base and converting it to a thymine or guanine, respectively. Base editors thus avoid DNA damage through DSBs, but have a restricted repertoire of point mutations.

### Methods for hematopoietic differentiation of human iPSCs

**Fig. 1.**
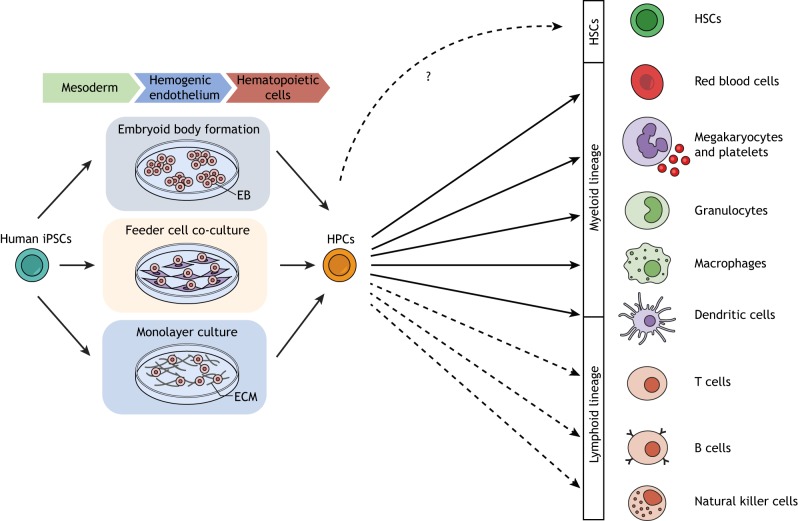
**Methods of hematopoietic differentiation of iPSCs.** There are three main culture methods for the hematopoietic differentiation of hPSCs: formation of embryoid bodies (EBs; 3D aggregates of hPSCs forced to remain in suspension culture); co-cultures with stromal feeder cells (most commonly the murine BM OP9 line); and monolayer cultures of extracellular matrix (ECM). A variation that generates EBs of controlled size is the ‘spin-EB’ method, based on EB formation by dissociation and reaggregation by centrifugation. EB-based and monolayer protocols generally afford more control over the steps of the differentiation process, which involve the addition of growth factors and cytokines in a specific order and dose. This step-wise procedure at first forces iPSCs to exit pluripotency and commit to a mesoderm fate. This is followed by the induction of hemogenic endothelium and, subsequently, by the specification of hematopoietic lineages. Current protocols of *in vitro* hPSC directed differentiation likely do not generate true HSCs. Cells of both myeloid (red blood cells, platelets, granulocytes, macrophages, dendritic cells) and lymphoid lineages (T, B and natural killer cells) can be generated by administering the appropriate cocktail of cytokines and growth factors and, in some cases, by co-cultures with stromal cells.

Effectively, all disease modeling applications involve differentiation of the iPSC lines of interest, along with their controls, to the cell type in which the disease manifests. Although mutations associated with blood diseases rarely cause molecular and phenotypic changes at the pluripotent state, this is rather the exception. Typically, the appropriate hematopoietic cell type needs to be generated and studied by a process of directed *in vitro* differentiation, in which iPSCs are induced to exit the pluripotent state and, first, commit to a mesoderm fate. This is followed by the induction of hemogenic endothelium – a specialized form of endothelium that gives rise to blood during development – and, subsequently, by the specification of the hematopoietic lineage (Fig. 1) (Ackermann et al., 2015; Sturgeon et al., 2013; Yoder, 2014).

#### Development of the hematopoietic system

Protocols for hematopoietic differentiation attempt to mimic the developmental processes that give rise to the hematopoietic system during ontogeny. Our understanding of the latter thus both informs and limits our ability to derive hematopoietic lineages that faithfully recapitulate their primary counterparts. During mammalian embryogenesis, the hematopoietic system is established in successive waves that each gives rise to distinct types of hematopoietic cells. The first two waves originate from an extra-embryonic site, the yolk sac. The first wave gives rise predominantly to erythrocytes with distinct characteristics from those that appear later in life, as well as to some macrophage and megakaryocyte progenitors (Palis et al., 1999). The second wave generates erythro-myeloid progenitors (EMPs) that produce most myeloid lineages, as well as T and B lymphocyte progenitors (Chen et al., 2011; Palis et al., 1999; Yoshimoto et al., 2011, 2012). The third wave emerges in the embryo proper, first within the major arteries, including the dorsal aorta (part of the aorta-gonad-mesonephros region), subsequently in the fetal liver (FL) and finally in the BM. Only the third wave generates hematopoietic stem cells (HSCs) with multilineage potential and the potential to engraft adult recipients. Most of our knowledge of this ontogeny comes from mouse studies and its equivalency to human hematopoiesis is not always established. In particular, crucially for hPSC-derived hematopoiesis, the existence of the second wave has not been formally demonstrated in humans. Furthermore, the nomenclature is often confusing. Although the first wave and its products are generally referred to as ‘primitive’ and the third as ‘definitive’, the second wave can be characterized as either primitive or definitive in the literature, depending on how different researchers define these terms (Lacaud and Kouskoff, 2017; Ivanovs et al., 2017).

#### hPSC-derived hematopoiesis

Although the different waves of hematopoiesis are temporally and spatially distinct during embryonic development *in vivo*, the current protocols for *in vitro* hematopoietic differentiation of hPSCs fail to clearly separate these developmental stages (Choi et al., 2012; Pearson et al., 2015; Zambidis et al., 2005). This loss of spatiotemporal separation is further complicated by the fact that no clear markers or assays exist to identify all the different cell types originating from different waves, in particular the EMPs from FL-derived hematopoiesis (Lacaud and Kouskoff, 2017; Ivanovs et al., 2017). Furthermore, although a matter of earlier debate, it is now clear that all developmental waves of hematopoiesis happen via an endothelial intermediate undergoing an endothelial-to-hematopoietic transition (EHT), as also occurs in hPSC-derived hematopoietic cultures (Lacaud and Kouskoff, 2017; Ivanovs et al., 2017). Whereas earlier protocols yielded mostly primitive hematopoiesis, the Keller lab later demonstrated the importance of early mesoderm patterning through activin inhibition or WNT stimulation to tilt the balance towards definitive over primitive hematopoietic lineages (Kennedy et al., 2012; Sturgeon et al., 2014). Whether these cells recapitulate EMPs or belong to the third wave of hematopoiesis with the potential to generate HSCs remains uncertain. However, there is wide consensus that current protocols of *in vitro* hPSC directed differentiation likely do not generate true HSCs, at least not efficiently enough to be detectable. Indeed, numerous attempts have failed to demonstrate multilineage engraftment ability of hPSC-derived hematopoiesis, and *in vitro* multilineage potential that includes the lymphoid lineage has not been demonstrated at the clonal level (Vo and Daley, 2015). This appears to be a limitation of current differentiation protocols and not an indication of a lack of HSC potential of hPSCs, as HSCs with long-term repopulating capability can be generated *in vivo* from human iPSC-derived teratomas (Amabile et al., 2013; Suzuki et al., 2013).

#### Hematopoietic lineages produced from hPSCs

Regardless of these technical limitations, *in vitro* hematopoietic differentiation of hPSCs has progressed with leaps over the past few years, and the current differentiation protocols have vastly improved efficiency, yield and reproducibility compared to those utilized in the (not so distant) past. There are three main culture methods for the hematopoietic differentiation of hPSCs: formation of embryoid bodies (EBs), co-cultures with stromal feeder cells (most commonly the murine BM OP9 line) and monolayer cultures (Kennedy et al., 2012; Rowe et al., 2016; Slukvin, 2013; Sturgeon et al., 2013) (Fig. 1). A variation that generates EBs of controlled size is the ‘spin-EB’ method, based on EB formation by dissociation, and reaggregation by centrifugation (Ng et al., 2005, 2008).

As mentioned previously, iPSC hematopoietic differentiation cultures mainly yield hematopoietic progenitor cells (HPCs) rather than HSCs. These can be further differentiated down different hematopoietic lineages using appropriate media and cytokines. The various assays and culture conditions for terminal differentiation that have been developed for *ex vivo* cultured human CD34^+^ hematopoietic stem/progenitor cells (HSPCs) over the years generally serve as templates for the generation of hPSC-derived HPCs. However, the terminal maturation and yield of lineage differentiation cultures originating from hPSC-HPCs are currently significantly lower than those obtained from *ex vivo* human CD34^+^ HSPCs. Red blood cells generated from hPSCs show limited enucleation and express embryonic and fetal hemoglobins, with little or no adult β-globin expression (Chang et al., 2011; Migliaccio et al., 2012; Dias et al., 2011; Ma et al., 2008). Similarly, hPSC-derived megakaryocytes yield relatively low numbers of platelets (Borst et al., 2017; Feng et al., 2014; Liu et al., 2015; Nakamura et al., 2014; Sullivan et al., 2014; Takayama et al., 2008), and hPSC differentiation towards granulocytes, macrophages and dendritic cells (DCs) also results in lower yields of mature cells (Choi et al., 2009; Lachmann et al., 2015; Senju et al., 2011). The generation of lymphoid lineages from both primary CD34^+^ HSPCs and hPSC-derived HPCs is much less efficient and more laborious than that of myeloid lineages (Ackermann et al., 2015; Wahlster and Daley, 2016). T cells are commonly generated *in vitro* from primary CD34^+^ HSPCs or hPSC-derived HPCs through several weeks of monolayer co-culture on OP9 stroma engineered to express the Notch ligands DLL1 or DLL4, with the addition of cytokines. Maturation of hPSC derivatives into T cells is much more limited than that of primary CD34^+^ HSPC derivatives (Montel-Hagen and Crooks, 2018; Schmitt and Zuniga-Pflucker, 2002). Reports of successful B cell generation are even fewer. French et al. reported the generation of CD19^+^/CD10^+^ B cells with VDJ recombination and cell-surface IgM expression from iPSC-HPCs cultured on MS5 stroma (French et al., 2015). Mature natural killer (NK) cells have, however, recently been efficiently generated from human iPSCs, by co-culture of CD34^+^/CD45^+^ HPCs on the murine stromal cell line EL08-1D2 with the addition of SCF, FLT3L, IL-15, IL-7 and IL-3 (Li et al., 2018).

## iPSC models of hematologic disorders

**Fig. 2.**
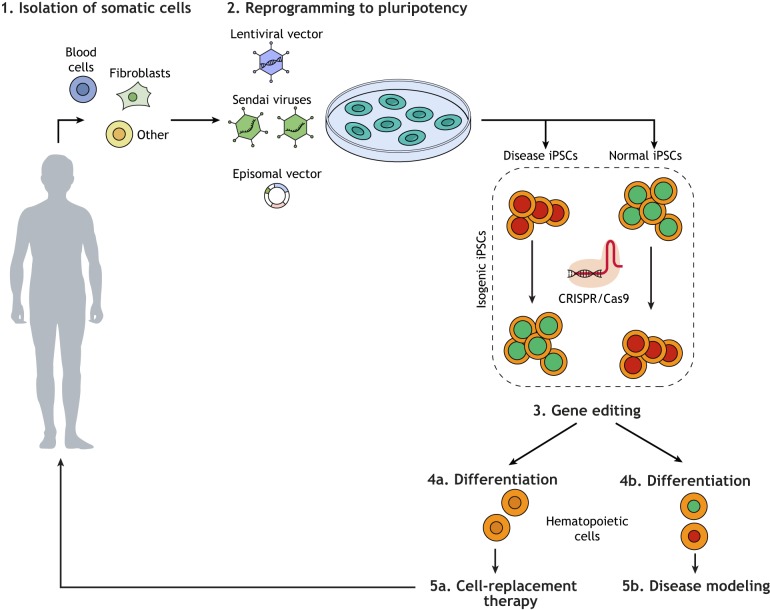
**Overview of iPSC applications in biomedical research.** General scheme of experimental workflow of iPSC use for modeling diseases or for the derivation of cell products for cell therapy. First, somatic cells of various sources (e.g. blood cells, fibroblasts) are isolated from the patient (1). They are then reprogrammed to iPSCs with reprogramming factors using various delivery methods (lentiviral vectors, episomal vectors or Sendai viruses) (2). Isogenic pairs or iPSCs can be generated through gene editing to either correct the mutation in disease iPSCs or to introduce it in normal iPSCs (3). Upon *in vitro* differentiation into the appropriate cell type (4a and 4b) the cells can be used for either cell-replacement therapy (5a) or disease modeling (5b) applications.

The applications of iPSC models of human disease fall under two broad categories: investigation of disease mechanisms towards classic drug development and development of gene and cell therapy strategies (Fig. 2). In the first, iPSC lines serve as research reagents towards therapeutic discoveries. In the second, they serve as the therapeutic agents themselves. Here, we will focus on two areas of disease modeling for blood disorders: the preclinical modeling of gene therapy of inherited blood diseases and the modeling of blood cancers. Other promising applications, such as iPSC-derived blood transfusion products (red blood cells, platelets) or immune cells for immunotherapy (T cells, NK cells, DCs), will not be covered here (Kaufman, 2009).

### iPSC models of inherited monogenic blood disorders

Several iPSC models of inherited monogenic blood diseases have been developed as preclinical models of gene and cell therapy approaches, under a general scheme which includes the following steps: (1) isolation of a patient's somatic cells; (2) reprogramming into iPSCs; (3) genetic correction of the underlying mutation; (4) *in vitro* differentiation into the appropriate cell type; (5) autologous transplantation of the corrected cells (Fig. 2). A variety of approaches to gene therapy, through either addition of the defective gene or gene editing of the mutation, has been described (Table 1).

**Table 1.**
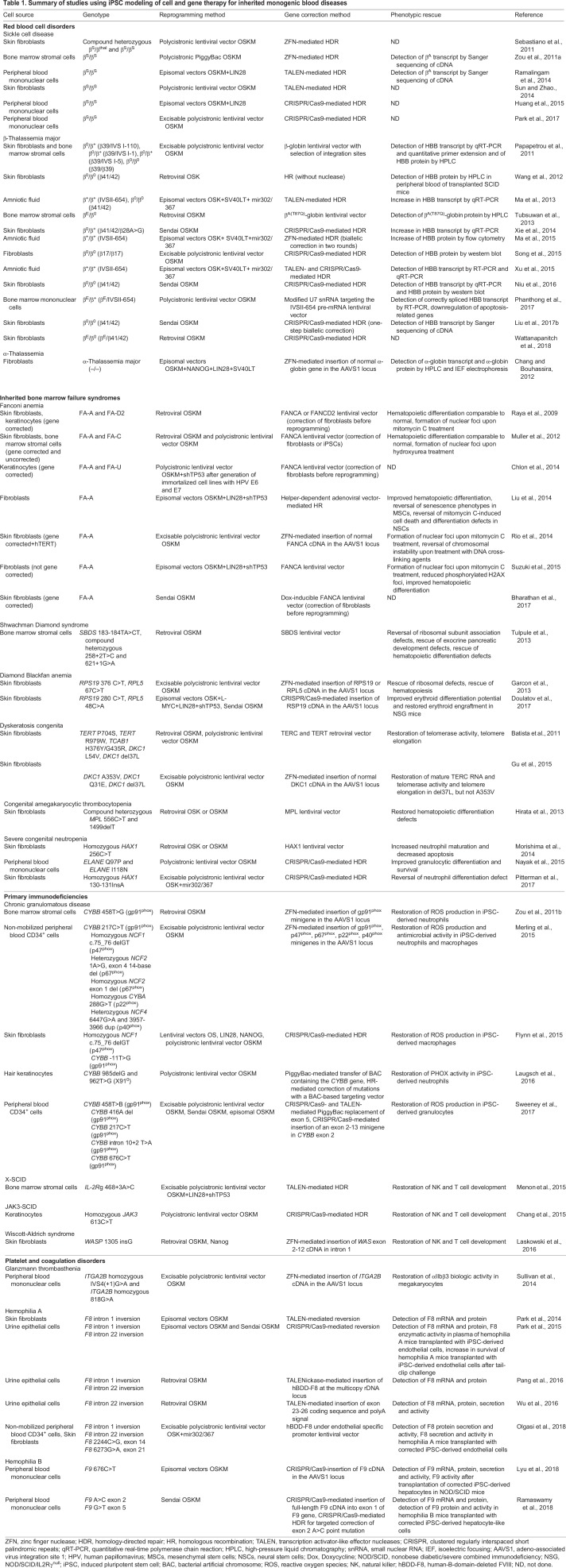
**Summary of studies using iPSC modeling of cell and gene therapy for inherited monogenic blood diseases**

#### Hemoglobinopathies: thalassemias and sickle cell disease

The hemoglobinopathies are the most common and extensively studied inherited monogenic disorders in the human population. They include thalassemias, which are caused by mutations that severely reduce or completely abolish globin gene expression, and diseases caused by mutations that result in pathologic structural variants of hemoglobin, with sickle cell disease (SCD) being the most prominent. The first ever proof-of-principle study of gene and cell therapy with autologous iPSCs used a SCD model, albeit in the mouse (Hanna et al., 2007). Since then, several groups have generated and corrected iPSCs from patients with SCD (Huang et al., 2015; Park et al., 2017; Ramalingam et al., 2014; Sebastiano et al., 2011; Sun and Zhao, 2014; Ye et al., 2009a; Zou et al., 2011a) and β-thalassemia of various genotypes (β^0^/β^0^, β^0^/β^+^, β^Ε^/β^0^, β^S^/β^+^, β^S^/β^S^) (Fan et al., 2012; Liu et al., 2017b; Ma et al., 2013, 2015; Niu et al., 2016; Papapetrou et al., 2011; Phanthong et al., 2017; Song et al., 2015; Tubsuwan et al., 2013; Wang et al., 2012; Wattanapanitch et al., 2018; Xie et al., 2014, 2015; Ye et al., 2009a). The first human iPSC gene and cell therapy study used a lentiviral vector similar to those that have since shown therapeutic benefit in clinical trials of autologous HSC gene therapy for β-thalassemia and SCD (Boulad et al., 2014; Thompson et al., 2018; Papapetrou et al., 2011). A major concern with gene therapy in HSCs is insertional mutagenesis by the therapeutic vectors which integrate into the genome randomly and can drive malignant transformation. Because, unlike HSCs, iPSCs can be cloned and selected, this study demonstrated the feasibility of excluding clones with potentially harmful lentiviral insertions and selecting clones with vector integrations in ‘safe harbor’ sites of the human genome to avoid insertional leukemogenesis (Papapetrou et al., 2011; Papapetrou and Schambach, 2016; Sadelain et al., 2011).

As gene editing tools evolved, ‘*in-situ*’ mutation correction approaches using HDR and nucleases (ZFN, TALEN, CRISPR-Cas9) were successfully used by several groups to correct SCD and β-thalassemia patient-derived iPSCs (Sebastiano et al., 2011; Li et al., 2011a; Huang et al., 2015; Liu et al., 2017b; Ma et al., 2013, 2015; Niu et al., 2016; Park et al., 2017; Ramalingam et al., 2014; Sun and Zhao, 2014; Wattanapanitch et al., 2018; Xie et al., 2014; Zou et al., 2011a; Song et al., 2015; Xu et al., 2015). Some studies achieved biallelic correction (Liu et al., 2017b; Ma et al., 2015), although in almost all cases of SCD and thalassemias, monoallelic correction of the mutation would be sufficient for phenotypic correction, as heterozygous status confers essentially normal erythropoiesis. Unfortunately, the developmental immaturity of iPSC-derived erythropoiesis did not allow any of these gene editing studies to convincingly show expression of β-globin from the corrected locus or phenotypic correction, as iPSC-derived erythroid cells expressed predominantly embryonic and fetal globins, and almost no endogenous β-globin. Although SCD is caused by a single point mutation, a variety of mutations encompassing the entire *HBB* gene cause β-thalassemia, rendering the development of autologous gene therapy by gene editing rather impractical. The same applies to α-thalassemia, which is most often caused by large deletions that cannot easily be corrected by HDR. In these cases, addition of a normal globin gene copy with selection of insertion sites or targeted insertion to a predetermined safe harbor site, such as AAVS1, can provide an alternative approach (Chang and Bouhassira, 2012; Papapetrou et al., 2011).

#### Inherited bone marrow failure syndromes

Inherited bone marrow failure syndromes (IBMFS) are another group of inherited blood disorders that are characterized by the decreased production of mature blood cells of one or more lineages, often with a predisposition to leukemia development. iPSC models of several IBMFS have been created, including Fanconi anemia (FA) (Bharathan et al., 2017; Chlon et al., 2014; Liu et al., 2014; Muller et al., 2012; Raya et al., 2009; Rio et al., 2014; Suzuki et al., 2015; Yung et al., 2013; Navarro et al., 2014), Shwachman Diamond syndrome (Tulpule et al., 2013), Diamond Blackfan anemia (DBA) (Doulatov et al., 2017; Garcon et al., 2013), dyskeratosis congenita (DC) (Agarwal et al., 2010; Batista et al., 2011; Gu et al., 2015; Jose et al., 2018), amegakaryocytic thrombocytopenia (Hirata et al., 2013) and severe congenital neutropenia (Morishima et al., 2014; Nayak et al., 2015; Pittermann et al., 2017; Hiramoto et al., 2013). Because IBMFS are characterized by a decline in the number and function of HSCs, iPSC-based cell therapy approaches, bypassing the need to harvest a patient's own HSCs, could hold promise for the future treatment of these diseases.

FA is caused by loss-of-function mutations in a number of genes of the FA pathway, which controls DNA damage repair, particularly that caused by DNA cross-linking agents (Kottemann and Smogorzewska, 2013). FA patients are susceptible to the development of various cancers. Efforts to reprogram somatic cells from FA patients revealed that FA cells are highly refractory to reprogramming (Bharathan et al., 2017; Chlon et al., 2014; Raya et al., 2009; Rio et al., 2014; Yung et al., 2013; Muller et al., 2012). This is perhaps the most dramatic example of a human disease-related pathway with such a profound effect on the reprogramming ability of the cells. In most studies, reprogramming was only possible after lentiviral gene complementation of *FANCA* or *FANCD2* in the patient fibroblasts (Bharathan et al., 2017; Chlon et al., 2014; Muller et al., 2012; Raya et al., 2009; Rio et al., 2014). In cases in which iPSCs could be generated from uncorrected FA cells, the efficiency was very low and some, but not all, of these iPSCs had abnormal karyotypes or other genome instability marks (Muller et al., 2012; Liu et al., 2014). Given these results, the generation of autologous gene-corrected iPSC-derived cells for FA patients in the future will necessitate extensive quality control of their genome and may require gene correction before reprogramming.

DBA primarily affects the erythroid lineage and is most often caused by heterozygous loss-of-function mutations in ribosomal protein genes, resulting in their haploinsufficiency. DBA-iPSCs with *RPS19* and *RPL5* mutations were generated and shown to have multilineage defects that were rescued by gene correction (Garcon et al., 2013). It is not clear why a multilineage *in vitro* phenotype was observed, as DBA pathology is largely restricted to the erythroid lineage. It may reflect more subtle defects that may be present in other lineages in the patients but only manifest under stress conditions. Doulatov et al. generated expandable HPCs from DBA-iPSCs through inducible expression of 5 factors (Table 1), thus circumventing restrictions of cell numbers, enabling a small-molecule screen. This led to the identification of SMER28, a positive regulator of autophagy, which rescued erythroid differentiation (Doulatov et al., 2017). This study proposed a potential new role for autophagy in the disease pathogenesis and a potential new therapeutic target.

DC is a rare IBMFD that is characterized by short telomeres and caused by mutations in telomere maintenance genes, most commonly in the telomerase genes (*TERC*, *TERT*) and the dyskerin gene (*DKC1*). iPSC models of DC have generated somewhat conflicting results, owing to the facts that different DC mutations confer variable disease severity and that reprogramming of fibroblasts – both DC and normal – to iPSCs results in telomerase reactivation and significant telomere elongation. Whereas one study reported telomerase upregulation and restoration of telomere length to normal levels upon reprogramming (Agarwal et al., 2010), others found that iPSCs with *TERT* or *TERC* mutations had reduced telomere elongation compared to normal cells (Batista et al., 2011; Gu et al., 2015; Winkler et al., 2013). Moreover, extended culture of iPSCs with *DKC1* mutations – causing a severe form of DC – led to progressive telomere shortening that was rescued by TERT or TERC overexpression (Batista et al., 2011).

#### Primary immunodeficiencies

A few iPSC models of gene therapy for primary immunodeficiencies – genetic disorders of impaired immunity against pathogens – have been generated, including for severe combined immunodeficiency (SCID; X-linked, RAG1-SCID and ADA-SCID), chronic granulomatous disease (CGD) and Wiscott-Aldrich syndrome (WAS) (Brauer et al., 2016; Chang et al., 2015; Dreyer et al., 2015; Flynn et al., 2015; Ingrungruanglert et al., 2015; Jiang et al., 2012; Laskowski et al., 2016; Laugsch et al., 2016; Menon et al., 2015; Merling et al., 2015; Park et al., 2008a; Sweeney et al., 2017; Zou et al., 2011b). CGD is caused by mutations in genes coding for any of the subunits of the enzyme phagocyte NADPH oxidase (PHOX), which is required for phagocytes (i.e. neutrophils and macrophages) to produce reactive oxygen species (ROS) to destroy bacteria. Genetic correction of CGD-iPSCs restored ROS production, phagocytosis and antimicrobial activity in iPSC-derived neutrophils and macrophages (Flynn et al., 2015; Laugsch et al., 2016; Merling et al., 2015; Sweeney et al., 2017; Zou et al., 2011b; Dreyer et al., 2015). Correction of SCID- and WAS-iPSCs rescued their defects in generation of NK and T cells (Chang et al., 2015; Laskowski et al., 2016; Menon et al., 2015).

#### Hemophilias

Hemophilia A and B (HA, HB) are X-linked bleeding disorders that are caused by mutations in the *F8* and *F9* genes, respectively, encoding the blood coagulation factors VIII and IX. Almost half of all severe HA cases result from two large (140 kb and 600 kb) chromosomal inversions at introns 1 and 22 of *F8*, respectively, both of which have been modeled in iPSCs (Olgasi et al., 2018; Pang et al., 2016; Park et al., 2015, 2014; Wu et al., 2016). Researchers achieved correction through a number of strategies: reversion induced by nuclease (TALEN or CRISPR-Cas9) pairs targeting the two breakpoint junctions (Park et al., 2015, 2014); targeted insertion of multiple copies of a modified *F8* coding sequence (human B-domain-deleted, hBDD-F8) in the ribosomal DNA locus (Pang et al., 2016); ectopic expression of hBDD-F8 via a lentiviral vector (Olgasi et al., 2018); and, in a case of intron 22 inversion, TALEN- mediated insertion of the 627-bp coding sequence spanning exons 23-26 into the exon22/intron 22 junction (Wu et al., 2016). Unlike HA, HB is caused primarily by point mutations in *F9* and could be corrected in iPSC models using mutation-specific CRISPR-Cas9-mediated HDR, in addition to more universal approaches, such as CRISPR-Cas9-mediated insertion of a F9 cDNA into exon 1 of the mutant gene or into the AAVS1 safe harbor locus (Lyu et al., 2018; Ramaswamy et al., 2018). Although gene therapy for hemophilias using *in vivo* delivery approaches currently shows promising results in clinical testing, iPSC-based cell and gene therapy could provide an alternative approach in the future. At the very least, iPSCs carrying patient mutations can serve as faithful genetic preclinical models of gene correction.

### iPSC models of myeloid malignancies

Efforts to develop iPSC models of blood cancers have so far almost exclusively concentrated on malignancies of the myeloid lineage, primarily owing to the difficulty of generating lymphoid lineages from iPSCs. Myeloid malignancies are classified as myeloproliferative neoplasms (MPN), myelodysplastic syndromes (MDS), MDS/MPN overlap syndromes and acute myeloid leukemia (AML).

#### MPN

iPSC models of MPN were the first to be developed, likely reflecting the abundance and *ex vivo* growth ability of MPN cells. The majority of studies have focused on chronic myeloid leukemia (CML) and JAK2-mutant MPN (Hu et al., 2011; Gandre-Babbe et al., 2013; Kumano et al., 2012; Hosoi et al., 2014; Ye et al., 2014; Suknuntha et al., 2015; Sloma et al., 2017; Mulero-Navarro et al., 2015; Miyauchi et al., 2018; Amabile et al., 2015; Ye et al., 2009b). CML is driven by the *BCR-ABL* fusion oncogene and treatment with the tyrosine kinase inhibitor imatinib results in lasting remission for the vast majority of patients. Several groups showed that CML-iPSCs do not respond to treatment with imatinib at the pluripotent undifferentiated state, despite *BCR-ABL* expression, indicating that they are not dependent on *BCR-ABL* at this cellular state. In contrast, sensitivity to imatinib reappeared in *in vitro* differentiated CD34^+^ HSPCs and, particularly, in further differentiated CD34^−^/CD45^+^ hematopoietic cells (Carette et al., 2010; Kumano et al., 2012; Miyauchi et al., 2018; Bedel et al., 2013; Suknuntha et al., 2015). Amabile et al. found that reprogramming and subsequent differentiation of CML cells reduces their oncogenic potential because patient-derived CML-iPSCs could differentiate into mature erythroid and myeloid cells and did not transplant leukemia into mice, as did the primary CML cells. The authors attributed this at least in part to DNA demethylation, as they documented decreased methylation levels in CML-iPSCs and their hematopoietic progeny compared to primary CML cells (Amabile et al., 2015).

iPSCs from non-CML MPN, including essential thrombocytopenia, polycythemia vera and primary myelofibrosis, have also been generated by several groups (Saliba et al., 2013; Ye et al., 2014; Hosoi et al., 2014; Liu et al., 2017a; Takei et al., 2018; Ye et al., 2009b). iPSCs with the most common driver mutation found in non-CML MPNs, JAK2^V617F^, were generated (Hosoi et al., 2014; Saliba et al., 2013; Ye et al., 2014, 2009b), and HPCs derived from them recapitulated the characteristic hematopoietic cellular phenotype of cytokine-independent colony formation in methylcellulose (Saliba et al., 2013; Ye et al., 2014). Heterozygous and homozygous JAK2^V617F^-mutant iPSC lines could be derived from different patients and, in some cases, normal iPSCs could also be derived from the same patients (Hosoi et al., 2014; Ye et al., 2014). Researchers could also obtain iPSC lines with additional mutations acquired upon progression to AML (Gomez Limia et al., 2017; Saliba et al., 2013). The effects of JAK inhibitors used in the clinic were modeled in one of the studies: three JAK inhibitors, INCB018424 (Ruxolitinib), TG101348 (SAR302503) and CYT387, were tested on eythroblasts and HPCs derived from heterozygous and homozygous JAK2^V617F^-mutant iPSC lines. All three compounds inhibited differentiation and expansion of iPSC-derived erythroblasts, whereas they had no effect on the expansion of CD34^+^/CD45^+^ HPCs (Ye et al., 2014). These results may reflect the clinical observations that anemia is a common side effect of JAK inhibitors in clinical trials and that JAK inhibition is not sufficient to eliminate the disease. The other two MPN driver gene mutations, *MPL* and *CALR*, have also been captured in MPN patient-derived iPSCs and shown to model thrombopoietin-independent megakaryocytic colony formation (Liu et al., 2017a; Takei et al., 2018; Gomez Limia et al., 2017).

#### MDS/MPN overlap syndromes

Juvenile myelomonocytic leukemia (JMML) is a rare pediatric MDS/MPN overlap syndrome that is characterized by somatic mutations in *NF1*, *CBL*, *NRAS*, *KRAS* or *PTPN11* that result in the activation of RAS/MAPK or JAK/STAT signaling. iPSCs from a patient with a severe form of JMML caused by the PTPN11^E76K^ mutation recapitulated cytokine-independent colony growth and hypersensitivity to GM-SCF and were sensitive to the MEK inhibitor PD901 (Gandre-Babbe et al., 2013). Myeloid cells derived from JMML-iPSCs with PTPN11^E76K^ and the CBL^Y371H^ mutations showed differential signaling pathway activation and sensitivity to kinase inhibitors, specifically constitutive RAS/MAPK or JAK/STAT signaling, and sensitivity to MEK or JAK inhibitors, respectively (Gagne et al., 2018; Tasian et al., 2018). Mulero-Navarro et al. generated iPSCs from patients with germline *PTPN11* mutations (Noonan syndrome), and showed GM-CSF hypersensitivity and increased proliferation of PTPN11-mutant iPSC-derived myeloid cells (Mulero-Navarro et al., 2015). iPSCs derived from one adult patient with chronic myelomonocytic leukemia (CMML), another MDS/MPN overlap syndrome, recapitulated disease-related phenotypes *in vitro* and were used for drug testing (Taoka et al., 2018).

#### MDS

In contrast to MPN, MDS patient cells are much harder to obtain in sufficient numbers or to culture *ex vivo*, so cellular models of MDS are direly needed. Unfortunately, the reprogramming of MDS cells into iPSCs proved to be much more challenging than that of MPN cells. The first MDS-iPSCs were described by Kotini et al. and were generated from patients with MDS with chromosome 7q deletions (del7q) – a characteristic chromosomal abnormality in MDS (Kotini et al., 2015). Specifically, iPSCs with del7q and genetically matched normal iPSCs were derived from two MDS patients. Upon hematopoietic differentiation, del7q iPSCs gave rise to dramatically decreased numbers of CD34^+^/CD45^+^ myeloid progenitor cells and colonies in methylcellulose. Complementary iPSC lines were also generated by engineering del7q in normal iPSCs derived from the same patients. Using engineered del7q iPSC clones with deletions spanning different regions of chromosome 7q, as well as patient-derived del7q-MDS-iPSCs with spontaneous reversion of del7q (i.e. spontaneous duplication of the intact chr7), we were able to functionally map the MDS phenotypes to a region of 20 Mb spanning bands 7q32.3-7q36.1, the hemizygosity of which was sufficient to confer the loss of hematopoietic differentiation potential. We then selected candidate chromosome 7q haploinsufficient genes on the basis of reduced expression in del7q compared to matched normal iPSCs, built a lentiviral library of 75 candidate open reading frames with unique barcodes and performed a pooled screen for genes that would rescue the emergence of CD45^+^ HPCs from del(7q)-iPSCs. Four genes, *EZH2*, *LUC7L2*, *HIPK2* and *ATP6V0E2*, could be further validated by overexpression, as well as short hairpin RNA (shRNA) knockdown and monoallelic CRISPR-Cas9 inactivation (*EZH2*) in cord blood CD34^+^ cells. Interestingly, three of these four genes (*EZH2*, *LUC7L2* and *HIPK2*) have also been found to harbor monoallelic loss-of-function mutations in MDS, in further support of a function as haploinsufficient tumor suppressor genes in MDS.

Subsequently, additional MDS-iPSC lines were generated, including lines with chromosome 20q deletion and mutations of splicing factor (SF) genes (Kotini et al., 2017; Chang et al., 2018). Using CRISPR-Cas9, a series of isogenic iPSC lines harboring del7q, the SF mutation *SRSF2* P95L, both genetic lesions or none, were engineered. Each genetic lesion could be connected to specific phenotypes and drug responses (Chang et al., 2018). This study thus provided a new proof of principle for precision oncology applications using iPSC models. Despite the relative reprogramming refractoriness of MDS cells, combining high-efficiency reprogramming in clonality-preserving conditions with comprehensive mutational analyses of the starting cells and derivative iPSC lines to guide the provenance of the latter, allowed our group to generate a panel of iPSC lines that captured the stages of clonal evolution from clonal hematopoiesis to low-risk MDS, high-risk MDS and secondary AML (Kotini et al., 2017). Through a thorough phenotypic characterization of these iPSC lines upon hematopoietic differentiation, we identified phenotypes with disease-stage specificity or graded severity and constructed a phenotypic framework capturing the distinct stages of myeloid malignancy from preleukemia to AML through an MDS stage. Using this system and phenotypic map, we showed that we can model transitions between stages, including disease progression and reversal, by introducing and correcting, respectively, progression-associated mutations using CRISPR-Cas9. We also uncovered potential disease stage-specific effects of 5-azacytidine, a drug used as front-line therapy for MDS. Furthermore, transcriptome analyses of iPSC-derived CD34^+^ hematopoietic progenitors of all stages revealed gene expression signatures associated with disease progression and enrichment for gene sets derived from primary AML patient cells.

#### AML

AML cells appear to reprogram with even lower efficiency, presumably because of a higher burden of genetic and epigenetic abnormalities (Chao et al., 2017; Kotini et al., 2017; Hoffmann et al., 2016; Salci et al., 2015; Lee et al., 2017). Regardless, AML-iPSC lines have successfully been derived and studied. Amabile et al. observed no malignant phenotype in CML-iPSC-derived hematopoietic cells, as mentioned earlier (Amabile et al., 2015); however, both Chao et al. and Kotini et al. showed that, although AML-iPSCs appear to be roughly normal at the pluripotent state, clear leukemic features appear once they are differentiated in hematopoietic progenitors, and leukemic gene expression and epigenetic signatures are reestablished (Chao et al., 2017; Kotini et al., 2017). These differences in malignant phenotypes may be due to CML-iPSC phenotypes being more subtle, as AML is a much more aggressive disease than CML. Other technical reasons or interclonal variability may also be involved. Although AML-iPSC-derived hematopoietic cells clearly have leukemic phenotypes, reprogramming itself may restore some differentiation potential, as AML-iPSC-derived myeloid cells retained some capacity for differentiation – albeit much more limited compared to normal iPSCs (Kotini et al., 2017). Remarkably, both Chao et al. and Kotini et al. showed that hematopoietic cells from AML-iPSCs can engraft a serially transplantable lethal leukemia in NSG mice (Chao et al., 2017; Kotini et al., 2017). This is in striking contrast to the inability of normal iPSC-derived hematopoietic cells to repopulate immunodeficient mouse recipients. These studies were the first reports of robust engraftment of hematopoietic cells derived from hPSCs through directed differentiation without gene modification.

#### MDS/AML familial predisposition syndromes

Familial MDS/AML predisposition syndromes caused by inherited germline mutations have also been modeled in iPSCs. Familial platelet disorder (FPD) is characterized by thrombocytopenia, platelet dysfunction and predisposition to MDS/AML, and is caused by germline mutations of *RUNX1*, which can be either loss-of-function or dominant negative. Several groups derived iPSCs from different FPD pedigrees and showed megakaryocytic differentiation defects that were corrected upon gene targeting or overexpression of wild-type *RUNX1* (Antony-Debre et al., 2015; Connelly et al., 2014; Iizuka et al., 2015; Sakurai et al., 2014). Antony-Debre et al. used iPSC modeling to compare two different *RUNX1* mutations from two pedigrees, a loss-of-function mutation resulting in haploinsufficiency and a dominant negative R174Q mutation. They found that the latter had more profound phenotypic effects in addition to the megakaryocytic defects, which led them to suggest an effect for RUNX1 dosage in leukemic predisposition (Antony-Debre et al., 2015). Two groups generated iPSCs from patients with familial predisposition to MDS/AML with germline *GATA2* mutations. Kotini et al. found modest hematopoietic defects in a patient-derived GATA2-mutant iPSC line, consistent with a preleukemic state (Kotini et al., 2017). Jung et al. showed some reduction in hematopoietic differentiation potential, but they were not able to consistently attribute this phenotype to the mutational status (Jung et al., 2018). Importantly, Kotini et al. modeled leukemic progression by engineering additional genetic lesions, either CRISPR-Cas9-mediated inactivation of the second *GATA2* allele or chromosome 7q deletion, and showed phenotypic progression to low-risk and high-risk MDS, respectively (Kotini et al., 2017).

## Strengths and limitations of iPSC models compared to other disease modeling approaches

iPSCs are now increasingly used in basic and translational hematology research alongside models with longer track records. These include model organisms – mainly genetically engineered mouse models (GEMMs), zebrafish and non-human primates – and cellular models, mainly immortalized cell lines and primary patient cells. Patient-derived iPSCs occupy a unique niche in disease modeling in that they maintain a high degree of disease relevance, as patient-derived cells, and at the same time – unlike primary patient cells – enable rigorous experimentation. A major strength of iPSCs is that they provide genetically precise and faithful models because they retain the patient's genome. They thus allow interrogation of disease genetics in a physiologic genomic context. At the same time, they offer the possibility to study this in the most appropriate cellular context, as they can theoretically produce any cell type of the human body. This property also endows iPSC disease modeling with a unique capability, which is the interrogation of the contribution of cell type-specific factors to the development of a given disease and the interplay between disease genetics and epigenetics, as determined by cell type-specific gene expression programs and chromatin landscapes. For example, the requirement of a specific cell type for a given cancer-causing lesion to result in malignant transformation was shown in AML (Chao et al., 2017; Kotini et al., 2017) and can be investigated in other cancers in the future. In cases in which the disease-initiating cell is unknown, iPSC models may aid its identification. In addition, in diseases in which the interaction between diverse cell types – for example, stromal, endothelial or immune cells – is crucial to the pathogenesis, these relationships can be dissected and the contribution of each cell type investigated, as different cell types can be generated and purified from iPSCs through *in vitro* differentiation and added in co-cultures. Whereas cell-cell interactions can also be studied in *in vivo* models, these often require complex reporter and lineage tracing approaches.

Tools and principles developed for GEMMs can readily be applied to human iPSC models (Hockemeyer and Jaenisch, 2016). Whereas both the mouse and zebrafish are particularly good for reverse genetics studies, iPSCs capture intact human genomes, which additionally allows for the modeling of complex genotypes, such as structural chromosomal abnormalities, and preserves the genetic background of the patient with all potential – but as yet unidentified – genetic modifiers of disease. In particular, iPSCs can model large-scale chromosomal abnormalities, such as deletions, inversions and translocations, for which model organisms are inadequate because of limited conservation of synteny (Kotini et al., 2015). Likewise, modeling mutations affecting splicing or intronic and non-coding regions of the genome in other organisms is often not possible because of limited conservation. Similarly to the mouse and fish, iPSCs are well-suited for studying development and its disorders. Also, similarly to the fish, but not the mouse, they provide good platforms for medium- to high-throughput experiments, as unlimited cell numbers can be generated from them. However, this may not hold true for several applications requiring iPSC-derived cell types, as differentiation protocols have limits of scale. Considering cost, timelines and expertise required, the numbers of differentiated cells that can be obtained from iPSC models are significantly lower than the throughput that can be achieved with immortalized cell lines. As a tradeoff, iPSCs have a much higher degree of genetic stability, and provide both patient relevance and, crucially, availability of isogenic normal controls.

iPSC models also have limitations. Some are technical and may be overcome in the future, whereas others are intrinsic to iPSC models. First, although iPSCs, as human cells, benefit from an abundance of available research tools, such as a well-annotated genome, cell-surface markers and antibodies, which may not be available to model organisms like the fish (Avagyan and Zon, 2016), the iPSC field is just entering its second decade and thus iPSC-specific tools and expertise are still limited. Although iPSC lines from a variety of genetic disorders have been generated, these are not necessarily freely available or well-characterized enough to be useful to the broader community. Many more mutant lines, ideally with isogenic controls, as well as reporter lines will be needed, together with repositories that handle their distribution. Other issues, often related to user inexperience, are iPSC line-to-line variability in phenotypic readouts, and the acquisition of karyotypic or other genetic abnormalities over time in culture (Liang and Zhang, 2013). Inter-line variability can be due to epigenetic or genetic differences that go unnoticed, and is often exacerbated by suboptimal maintenance culture and differentiation procedures, for example by the introduction of bottlenecks, strong selection pressures or inefficient differentiation protocols that yield highly variable cell products. The accumulation of genetic abnormalities over time by cultured cells is a well-documented and ultimately unavoidable phenomenon, but can largely be mitigated by close monitoring and maintenance of early-passage cultures. Earlier concerns that reprogramming is per se mutagenic or that established iPSCs are inherently genetically unstable have now been debunked (Mayshar et al., 2010; Laurent et al., 2011; Peterson and Loring, 2014; Liang and Zhang, 2013; Young et al., 2012; Abyzov et al., 2012). Although iPSCs are exceptionally well-suited for mechanistic studies, they are at the same time reductionist models that do not capture all disease components the way whole organisms do. In addition, iPSCs have the potential to differentiate into all cell types, but the equivalency of *in vitro*-derived cells to their primary counterparts is currently poorly characterized. iPSC-derived cells may have impaired ability for terminal differentiation and likely retain some degree of developmental immaturity, i.e. they more closely resemble fetal or embryonic rather than adult cell types. Because of this, as with whole-organism models, studying disease-causing gene mutations is complicated by the developmental effects of these mutations, which may need to be overcome by engineering conditional alleles. Finally, as previously discussed, a current major limitation of iPSC models of blood disorders is the inability to derive engraftable HSCs (Vo and Daley, 2015).

### Outlook and future perspectives

In conclusion, there is no one-beats-all model, but rather a fit-for-purpose model for a given application. For example, iPSCs are excellent models to study the molecular mechanisms of specific disease-causing mutations, but are not great for studying complex diseases that manifest in adulthood or older age and involve interactions between multiple cell types. Limitations aside, iPSC models, together with other human models such as organoid cultures, are increasingly moving to the forefront of biomedical research and being used as primary discovery – as opposed to validation – tools.

Over the past decade, iPSC models have found increasing applications for the study of human disease. With their unique strengths, they have so far mainly complemented other models and confirmed or extended findings. Moving forward, technological advances will likely extend the capabilities of iPSC models, for example by incorporating microenvironmental components, such as stromal cells or mechanical forces (Ito et al., 2018) and the generation of organoid and other types of 3D culture from iPSCs mimicking the BM niche. Crucially, the generation of engraftable iPSC-derived hematopoiesis will greatly expand this system's capabilities in modeling both blood disorders and cell therapy approaches for their treatment. In the shorter term, more studies using iPSCs as primary tools will aid the building of trust and expertise in the hematopoiesis community towards broader adoption of this modeling approach.

We have entered an era in which the use of patient-derived tissues in biomedical research is rapidly expanding and being incorporated in drug development by industry. To date, iPSC models have mostly recapitulated known findings from animal models or patient cells, but primary discoveries made with them imminently await.
